# Changes in maternal risk factors and their association with changes in cesarean sections in Norway between 1999 and 2016: A descriptive population-based registry study

**DOI:** 10.1371/journal.pmed.1003764

**Published:** 2021-09-03

**Authors:** Ingvild Hersoug Nedberg, Marzia Lazzerini, Ilaria Mariani, Kajsa Møllersen, Emanuelle Pessa Valente, Erik Eik Anda, Finn Egil Skjeldestad

**Affiliations:** 1 Department of Community Medicine, Faculty of Health Sciences, UiT The Arctic University of Norway, Tromsø, Norway; 2 Institute for Maternal and Child Health–IRCCS “Burlo Garofolo”, Trieste, Italy; Cambridge University, UNITED KINGDOM

## Abstract

**Background:**

Increases in the proportion of the population with increased likelihood of cesarean section (CS) have been postulated as a driving force behind the rise in CS rates worldwide. The aim of the study was to assess if changes in selected maternal risk factors for CS are associated with changes in CS births from 1999 to 2016 in Norway.

**Methods and findings:**

This national population-based registry study utilizes data from 1,055,006 births registered in the Norwegian Medical Birth Registry from 1999 to 2016. The following maternal risk factors for CS were included: nulliparous/≥35 years, multiparous/≥35 years, pregestational diabetes, gestational diabetes, hypertensive disorders, previous CS, assisted reproductive technology, and multiple births. The proportion of CS births in 1999 was used to predict the number of CS births in 2016. The observed and predicted numbers of CS births were compared to determine the number of excess CS births, before and after considering the selected risk factors, for all births, and for births stratified by 0, 1, or >1 of the selected risk factors. The proportion of CS births increased from 12.9% to 16.1% (+24.8%) during the study period. The proportion of births with 1 selected risk factor increased from 21.3% to 26.3% (+23.5%), while the proportion with >1 risk factor increased from 4.5% to 8.8% (+95.6%). Stratification by the presence of selected risk factors reduced the number of excess CS births observed in 2016 compared to 1999 by 67.9%. Study limitations include lack of access to other important maternal risk factors and only comparing the first and the last year of the study period.

**Conclusions:**

In this study, we observed that after an initial increase, proportions of CS births remained stable from 2005 to 2016. Instead, both the size of the risk population and the mean number of risk factors per birth continued to increase. We observed a possible association between the increase in size of risk population and the additional CS births observed in 2016 compared to 1999. The increase in size of risk population and the stable CS rate from 2005 and onward may indicate consistent adherence to obstetric evidence-based practice in Norway.

## Abstract 2

In a population-based study, Ingvild Nedberg and colleagues investigate the relationship between maternal risk factors and changes in rates of cesarean section births in Norway.

## Author summary

**Why was this study done?:**

- A general increase in maternal risk factors is thought to be an important part of the explanation for increasing cesarean section (CS) worldwide.
- Few studies examine a combination of maternal risk factors.

**What did the researchers do and find?:**

- We studied the relationship between CS and 8 maternal risk factors over time in Norway.
- We found that most of maternal risk factors increased continuously, proportions of CS births also increased initially, then remained stable from 2005 till 2016.
- We found that most of the increase in proportions of CS was associated with an increase in maternal risk factors.

**What do these findings mean?:**

- Norway is one of the few high-resource countries that has managed to keep CS rates low and stable.
- The stable proportions of CS births from 2005 onward, while the size of the risk population continued to increase, may indicate that Norwegian obstetrical providers have not been influenced by possible factors driving CS rates in other parts of the world.

## Introduction

In 2015, 21.1% of babies worldwide were born by cesarean section (CS), an annual increase of 3.7% from 2000 [1]. The explanations for this increase are multifaceted and imply clinical, cultural, economic, organizational, and psychosocial factors. In principle, CS should always be medically justified, due to the increased risk of morbidity it confers on mothers and newborns [2]. Increases in the proportion of the population with increased likelihood of CS—i.e., women with maternal risk factors for CS such as advanced maternal age [3], obesity [4], diabetes [5], and previous CS [6] have been postulated as an important contributor to increasing CS rates. These increases are not restricted to high-income countries, as the largest population increases in body mass index and gestational diabetes have taken place in low- and middle-income countries [7,8].

Case management of women with known risk factors for CS depends on available resources; organization of antenatal and obstetric care; how work is divided between obstetricians and midwives; the existence of and compliance with international, national, and hospital guidelines; and obstetric culture [9]. The large variations in rates of induced labor, operative vaginal deliveries (OVD), and CS observed around the world, and even within countries, suggest different solutions to similar clinical challenges in antenatal and obstetric care.

Norway has a tax-funded health system, and all maternal healthcare facilities are public. Women can choose to receive antenatal care by either a midwife, a general practitioner, or a combination of both, and the program consists of 8 consultations including one ultrasound screening. Maternal health facilities consist of 3 levels: free-standing midwifery units, local hospitals, and university hospitals. The percentage of midwife-attended home births have been stable at less than 0.5% in the last 2 decades and are not part of the public maternal health system. Women are screened in antenatal care on a set of criteria to assure selection to the right level of care [10]. Care and treatment of women during pregnancy and labor in Norway are based on the principle of lowest effective level of care necessary to achieve the best outcome for mothers and newborns [11]. The neonatal mortality in Norway decreased gradually from 2.8 to 1.4 per 1,000 live births from 1999 to 2016 [12]. National CS policies have remained fairly restrictive in the past decades [10]; women have the right to codetermination, and their wishes regarding choice of delivery method must be taken into consideration, but the final decision to perform a CS is taken by a gynecologist [13]. Midwives are the main caregivers for low-risk women in labor.

Much attention has been given to single maternal risk factors for CS and their impact on CS rates [3,14,15]. However, there is also a need to assess the combined effect of several risk factors. Maternal risk factors for CS can, to a certain extent, be modified by changes in lifestyle, political incentives, and provider practice. It is, therefore, of interest to describe the impact of multiple risk factors on CS rates to better understand the complexity of CS trends. The aims of this study are 3-fold: (i) to describe changes in the proportion of CS births in Norway from 1999 to 2016; (ii) to describe changes in maternal risk profiles for births in Norway from 1999 to 2016; and (iii) to assess if changes in maternal risk factors for CS are associated with changes in the proportion of CS births in Norway over the 18-year study period.

## Materials and methods

### Study design and study population

The Norwegian Medical Birth Registry (NMBR) was established in 1967 and collects data throughout pregnancy, birth, and the postpartum period, including sociodemographic information on parents, maternal prepregnancy morbidity, pregnancy-related conditions and diseases, birth complications, and newborn outcomes. Registration in the NMBR is mandated by law. Attending midwives enter information into the NMBR using an online form; quality assurance measures are built into the form to ensure standard reporting of data. Predetermined violations of biological plausibility in the online form are handled by the operational staff at the NMBR (S1 Personal Communication). The present population-based registry study included all births registered in the NMBR from 1 January 1999 to 31 December 2016. Births, not women, are the denominator in this study. Births with missing information on gestational age and birth weight, as well as births with a gestational age <22 weeks or >44 weeks, and birth weight <500 g, were excluded. This study is reported as per the Strengthening the Reporting of Observational Studies in Epidemiology (STROBE) guideline (S1 STROBE Checklist) [16]. There was no prospective protocol or analysis plan for this study.

### Variables and data analysis

To present changes in population characteristics over time, the 18-year study period was divided into 6 time periods (1999 to 2001, 2002 to 2004, 2005 to 2007, 2008 to 2010, 2011 to 2013, and 2014 to 2016). We did not include births before 1999, since changes to the reporting of variables were implemented in 1998. Information was collected from the NMBR on parity (0, 1, 2, ≥3), maternal age (<20, 20 to 24, 25 to 29, 30 to 34, 35 to 39, ≥40 years), maternal morbidity (pregestational diabetes, gestational diabetes, chronic hypertension, gestational hypertension, preeclampsia, eclampsia, and hemolysis, elevated liver enzymes, low platelet count (HELLP) syndrome), previous CS (yes/no), assisted reproductive technology (ART) (yes/no), multiple births (yes/no), gestational age (22 to 28, 28 to 31, 32 to 36, 37 to 41, 42 to 44 weeks), onset of labor (spontaneous, induced, prelabor CS), and mode of delivery (spontaneous vaginal, OVD, CS). Diagnostic criteria in the NMBR of the selected risk factors did not change during the study period and are based on the International Classification of Diseases, Revision 10 [17], and recommendations from the Norwegian Society for Gynecology and Obstetrics [18]. There has been a demographic change in couples receiving ART with both a shorter duration of infertility before ART is offered, but also due to improved technology, women with more severe morbidity are included in this group [19]. Furthermore, we combined chronic hypertension, gestational hypertension, preeclampsia, eclampsia, and HELLP syndrome into a single variable called “hypertensive disorders,” due to the low number of cases apart from preeclampsia.

To describe changes in proportions of CS births in our study sample, we calculated the number of CS births each year divided by the total number of births each year. To select maternal risk factors for the study, we consulted the Norwegian obstetrical guidelines, which lists 10 risk factors for CS [20]. Of those, obesity was excluded since prepregnancy body mass index was introduced as a variable in 2008. Previous traumatic vaginal delivery, mental disorders, and birth anxiety were excluded since they are only registered as indications for CS, and not as prepregnancy or pregnancy-related conditions in NMBR. The prevalence of these conditions is therefore unknown. We also excluded breech presentation, since we considered it to be a fetal, not a maternal risk factor. We excluded induction of labor as we considered it a mediating variable between a risk factor and CS as an outcome. Thus, we considered advanced maternal age, diabetes mellitus, previous CS, and twins as maternal risk factors. Diabetes mellitus was divided into pregestational and gestational diabetes. Of the remaining maternal pre- and pregnancy-related conditions in NMBR, we included ART and hypertensive disorders since they are known to be risk factors for CS [21–23]. Advanced maternal age was defined as ≥35 years, divided into nulliparous and multiparous births, since this cutoff is used in national guidelines for national quality indicators and as a selection criterion to appropriate birth facility [13].

The outcome was CS overall, since the selected risk factors are associated with both prelabor and emergency CS.

To describe changes in maternal risk profiles over time, we calculated the proportion of births with a single risk factor and the proportion with a single risk factor in combination with any other of the selected risk factors by year. Based on previously published material on Norwegian CS rates [10,24], we compared the first and last year of the study period (1999 and 2016) after stratification by the presence of risk factors: births with 0 risk factors, births with 1 risk factor, and births with >1 risk factor. We then calculated the total number of births, vaginal births, and CS births for each group for both years. In addition, the mean number of risk factors for each birth was calculated for all births, vaginal births, and CS births for each study year.

To investigate whether changes in maternal risk factors for CS are associated with changes in the proportion of CS births over time, we estimated the predicted number of CS births in 2016 for all births, births with 0 risk factors, births with 1 risk factor, and births with >1 risk factor, based on the proportions of CS in 1999. Only the selected maternal risk factors in the study were considered in the calculation. The observed and the predicted numbers of CS births were then compared for each of the abovementioned groups before and after considering the selected risk factors, to determine the number of excess CS births. We stratified on the presence of maternal risk factors to assess if the selected risk factors could be associated with the change in CS over time. Posteriori, we calculated year-to-year percent change of observed CS overall and in the stratified groups to provide clarity for the reader.

In addition, we depicted the proportion of CS births for single selected risk factors and for single risk factors in combination with any other of the selected risk factor by year to investigate if any clear pattern emerged. Finally, we calculated the annual proportion of induced labors and the proportion of CS births among induced labors.

Analyses were performed using Stata/SE version 16.0 (Stata Corporation, College Station, TX).

### Ethical approval

The Regional Committee for Medical and Health Research Ethics South-East C (REK South-East 2010/3256) reviewed the study protocol with timely updates and approved the start and continuation of the study. The data are anonymized, adhering to Article 5 of the General Data Protection Regulation regulations. The research questions answered in this study were not part of the original study protocol.

## Results

**Fig 1:**
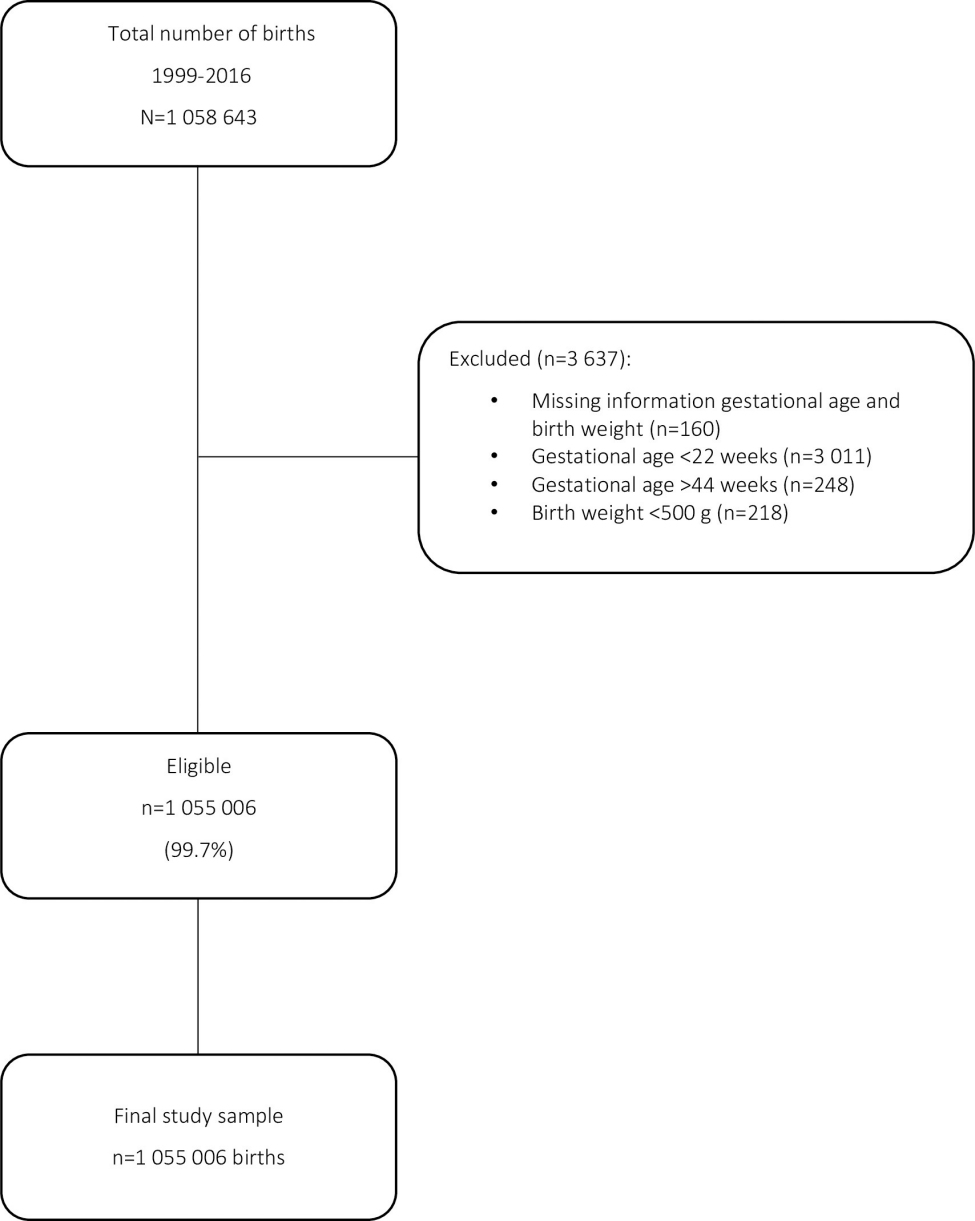
Selection of study sample.

After exclusions, the study sample comprised 1,055,006 births (Fig 1). Nulliparous women comprised 41.7%, women with a previous CS comprised 9.0%, and preterm births amounted to 6.1% of all births during the study period. Prelabor CS was performed in 7.7% of births. Spontaneous vaginal delivery occurred in 75.3% of births, OVD in 9.0%, and the average proportion of CS births for the whole time period was 15.7% (Table 1). The total proportion of missing data was low (0.7%).

**Table 1:** Characteristics of the study sample, stratified by 3-year time periods.

Study population	1999–2001 *(%*)	2002–2004 (*%*)	2005–2007 (*%*)	2008–2010 (*%*)	2011–2013 (*%*)	2014–2016 (*%*)	Total
**Parity**							
0	69,602 (*40*.*1*)	68,629 (*40*.*8*)	71,778 (*41*.*5*)	78,790 (*42*.*9*)	76,079 (*42*.*4*)	75,364 (*42*.*5*)	440,242 (*41*.*7*)
1	61,330 (*35*.*4*)	60,237 (*35*.*8*)	61,686 (*35*.*7*)	64,738 (*35*.*2*)	65,504 (*36*.*5*)	65,130 (*36*.*8*)	378,625 (*35*.*9*)
2	29,874 (*17*.*2*)	27,552 (*16*.*4*)	27,656 (*16*.*0*)	28,268 (*15*.*4*)	27,221 (*15*.*2*)	26,334 (*14*.*9*)	166,905 (*15*.*8*)
≥3	12,608 (*7*.*3*)	11,732 (*7*.*0*)	11,684 (*6*.*8*)	12,024 (*6*.*5*)	10,854 (*6*.*1*)	10,332 (*5*.*8*)	69,234 (*6*.*6*)
Missing	0	0 (*0*.*0*)	0 (*0*.*0*)	0 (*0*.*0*)	0 (*0*.*0*)	0 (*0*.*0*)	0 (*0*.*0*)
**Maternal age**							
<20 years	4,534 (*2*.*6*)	3,802 (*2*.*3*)	3,876 (*2*.*2*)	4,336 (*2*.*4*)	3,048 (*1*.*7*)	2,275 (*1*.*3*)	21,871 (*2*.*1*)
20–24 years	27,102 (*15*.*6*)	24,354 (*14*.*5*)	24,633 (*14*.*3*)	27,339 (*14*.*9*)	25,763 (*14*.*3*)	21,531 (*12*.*2*)	150,722 (*14*.*3*)
25–29 years	61,518 (*35*.*5*)	54,959 (*32*.*7*)	53,710 (*31*.*1*)	57,231 (*31*.*1*)	56,805 (*31*.*6*)	57,651 (*32*.*5*)	341,874 (*32*.*4*)
30–34 years	55,408 (*32*.*0*)	57,604 (*34*.*3*)	59,079 (*34*.*2*)	59,401 (*32*.*3*)	58,980 (*32*.*8*)	60,132 (*33*.*9*)	350,604 (*33*.*2*)
35–39 years	21,444 (*12*.*4*)	23,616 (*14*.*0*)	26,812 (*15*.*5*)	29,883 (*16*.*3*)	29,037 (*16*.*2*)	29,188 (*16*.*5*)	159,980 (*15*.*2*)
≥40 years	3,407 (*2*.*0*)	3,815 (*2*.*3*)	4,694 (*2*.*7*)	5,629 (*2*.*1*)	6,025 (*3*.*4*)	6,383 (*3*.*6*)	29,953 (*2*.*8*)
Missing	1 (*0*.*0*)	0 (*0*.*0*)	0 (*0*.*0*)	1 (*0*.*0*)	0 (*0*.*0*)	0 (*0*.*0*)	2 (*0*.*0*)
**Maternal morbidity**							
Pregestational diabetes	993 (*0*.*6*)	1,190 (*0*.*7*)	1,281 (*0*.*7*)	1,340 (*0*.*7*)	1,282 (*0*.*7*)	1,142 (*0*.*6*)	7,228 (*0*.*7*)
Gestational diabetes	1,391 (0.8)	1,441 (*0*.*9*)	1,985 (*1*.*2*)	3,014 (*1*.*6*)	4,645 (*2*.*6*)	8,172 (*4*.*6*)	20,648 (*2*.*0*)
Chronic hypertension	1,140 (*0*.*7*)	900 (*0*.*5*)	799 (*0*.*5*)	1,099 (*0*.*6*)	996 (*0*.*6*)	1,005 (*0*.*6*)	5,939 (*0*.*6*)
Gestational hypertension	2,365 (*1*.*4*)	3,073 *(1*.*8*)	3,352 (*1*.*9*)	3,434 (*1*.*9*)	3,281 (*1*.*8*)	2,730 (*1*.*5*)	18,235 (*1*.*7*)
Preeclampsia	7,392 (*4*.*3*)	6,691 (*4*.*0*)	6,439 (*3*.*7*)	6,137 (*3*.*3*)	5,102 (*2*.*8*)	4,728 *(2*.*7*)	36,489 (*3*.*5*)
Eclampsia	119 (*0*.*1*)	90 (*0*.*1*)	107 (*0*.*1*)	93 (*0*.*1*)	89 (*0*.*1*)	66 (*0*.*04*)	564 (*0*.*1*)
HELLP syndrome	325 (*0*.*2*)	288 (*0*.*2*	277 (*0*.*2*)	237 (*0*.*1*)	239 (*0*.*1*)	257 (*0*.*2*)	1,623 (*0*.*2*)
Hypertensive disorders*	8,677 (*5*.*0*)	8,292 *(4*.*9)*	8,103 (*4*.*7*)	8,464 (*4*.*6*)	7,593 *(4*.*2*)	6,906 (*3*.*9*)	48,125 (*4*.*6*)
**Previous CS** (1 or more)	13,844 (*8*.*0*)	14,140 (*8*.*4*)	15,391 (*8*.*9*)	16,992 (*9*.*2*)	17,019 (*9*.*5*)	17,401 (*9*.*8*)	94,787 (*9*.*0*)
**Assisted reproductive technology**	2,703 (*1*.*6*)	3,283 (*2*.*0*)	4,440 (*2*.*6*)	5,568 (3.0)	5,555 (3.1)	6,460 (3.7)	28,009 (*2*.*7*)
**Multiple births**	3,214 (*1*.*9*)	3,304 (*2*.*0*)	3,205 (*1*.*9*)	3,261 (*1*.*8*)	3,062 (*1*.*7*)	2,939 (*1*.*7*)	18,985 (*1*.*8*)
**Gestational age**							
22–27 weeks	827 (*0*.*5*)	729 (*0*.*4)*	719 *(0*.*4)*	693 (*0*.*4)*	663 (*0*.*4)*	702 (*0*.*4)*	4,333 (*0*.*4*)
28–31 weeks	1,262 (*0*.*7*)	1,180 *(0*.*7)*	1,134 (*0*.*7)*	1,149 (*0*.*6)*	1,040 (*0*.*6)*	998 (*0*.*6)*	6,763 (*0*.*6*)
32–36 weeks	9,064 (*5*.*2*)	9,042 *(5*.*4)*	9,116 *(5*.*3)*	9,106 (*5*.*0)*	8,480 (*4*.*7)*	8,322 (*4*.*7)*	53,130 (*5*.*0*)
37–41 weeks	146,808 (*84*.*7*)	143,778 (*85*.*5)*	148,301 (*85*.*8)*	160,099 *(87*.*1)*	161,274 (89.8)	159,826 (*90*.*2)*	920,086 (*87*.*2*)
42–44 weeks	14,406 (*8*.*3*)	12,210 *(7*.*3)*	12,346 (*7*.*1)*	11,298 (*6*.*2)*	7,388 (*4*.*1)*	6,761 *(3*.*8)*	64,409 (*6*.*1*)
Missing	1,047 (*0*.*6*)	1,211 (*0*.*7*)	1,188 (*0*.*8*)	1,475 (0.8)	813 (0.5)	551 (0.3)	6,285 (*0*.*6*)
**Onset of labor**							
Spontaneous	142,582 (*82*.*2*)	133,684 (*79*.*5*)	132,945 (*76*.*9*)	138,540 (*75*.*4*)	130,437 (*72*.*6*)	127,812 (*72*.*1*)	806,000 (*76*.*4*)
Induced	18,622 (*10*.*7*)	21,073 (*12*.*5*)	25,109 (*14*.*5*)	30,563 (*16*.*6*)	35,577 (*19*.8)	37,303 (*21*.*1*)	168,247 (*16*.*0*)
Prelabor CS	12,210 (*7*.*0*)	13,393 (*8*.*0*)	14,720 (*8*.*5*)	14,715 (*8*.*0*)	13,644 (*7*.*6*)	12,044 (*6*.*8*)	80,726 (*7*.*7*)
Missing	0 *(0*.*0*)	0 (*0*.*0)*	30 (*0*.*02*)	2 (*0*.*0*)	0 (*0*.*0*)	1 (*0*.*0*)	33 (*0*.*02*)
**Mode of delivery**							
Spontaneous vaginal	136,564 (*78*.*8*)	129,180 (*76*.*8*)	130,117 (*75*.*3*)	135,826 (*73*.*9*)	132,233 (*73*.*6*)	129,956 (*73*.*4*)	793,876 (*75*.*3*)
Operative vaginal	13,238 (*7*.*6*)	13,317 (*7*.*9*)	14,853 (*8*.*6*)	17,537 (*9*.*5*)	17,985 (*10*.*0*)	18,393 (*10*.*4*)	95,323 (*9*.*0*)
CS	23,612 (*13*.*6*)	25,653 (*15*.*3*)	27,834 (*16*.*1*)	30,457 (*16*.*6*)	29,440 (*16*.*4*)	28,811 (*16*.*3*)	165,807 (*15*.*7*)
Missing	0 *(0*.*0*)	0 (*0*.*0*)	0 (*0*.*0*)	0 (*0*.*0*)	0 (*0*.*0*)	0 (*0*.*0*)	0 (*0*.*0*)

CS, cesarean section; HELLP, hemolysis, elevated liver enzymes, low platelet count.

*Combined variable including gestational hypertension, chronic hypertension, preeclampsia, eclampsia, and HELLP syndrome.

**Fig 2:**
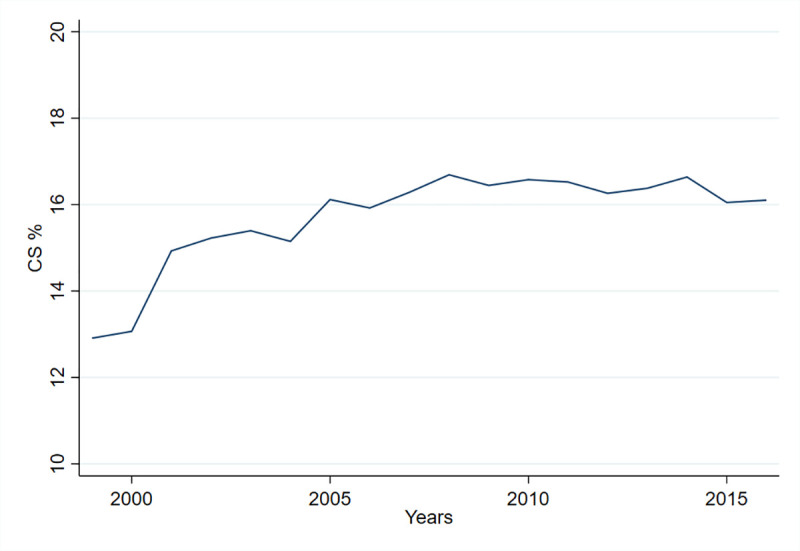
Proportions of CS in all births expressed as % per year, 1999–2016. The total proportion of births in Norway delivered by CS during the study period. CS, cesarean section.

The overall proportion of CS births in Norway increased from 12.9% in 1999 to 16.1% in 2016 (Fig 2), constituting a 24.8% increase (from 7,571 to 9,521 CS births). The largest yearly increase was seen between 2000 and 2001, when the proportion of CS births increased from 13.1% to 14.9%; from 2005 to the end of the study period, the proportion remained stable at 16% (±0.8%).

**Fig 3:**
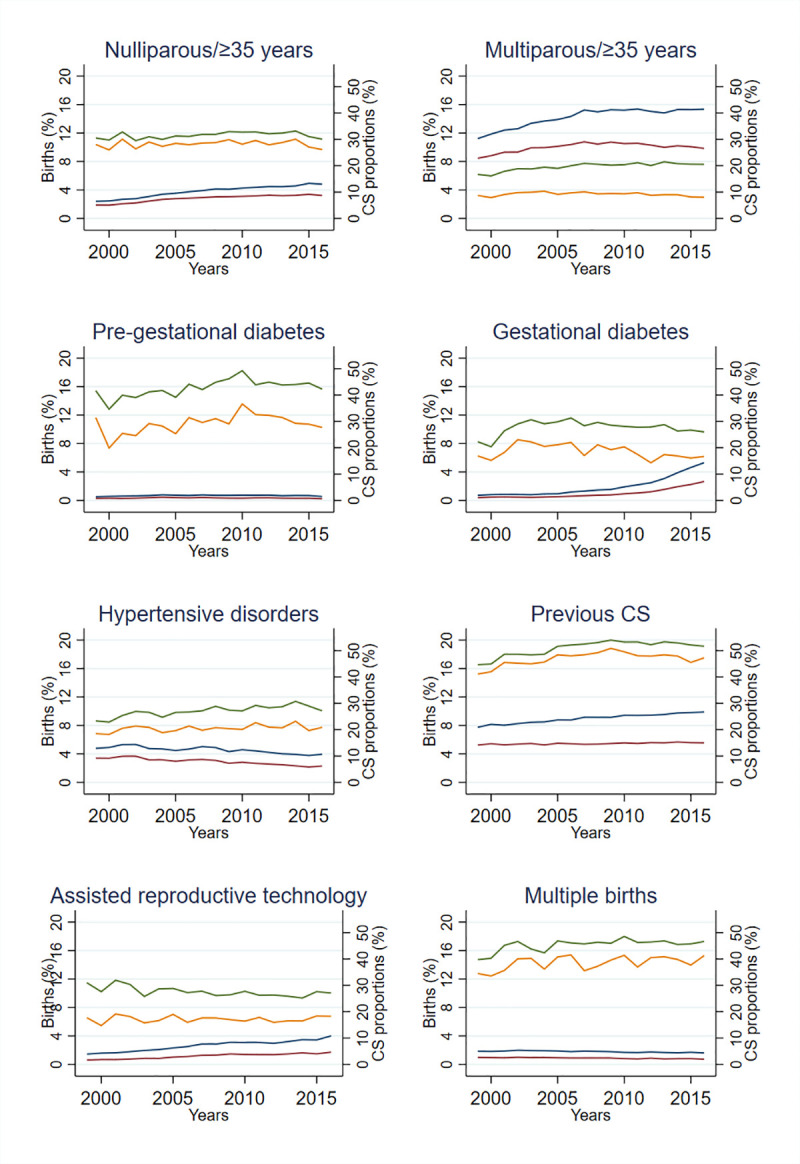
Each risk factor by proportion of births and corresponding CS births, by year 1999–2016 (%). Proportion of births with a single risk factor (red). Proportion of births with a single risk factor in combination with any other risk factor (blue). Proportion of CS births with a single risk factor (yellow). Proportion of CS births with a single risk factor in combination with any other risk factor (green). CS, cesarean section.

**Fig 4:**
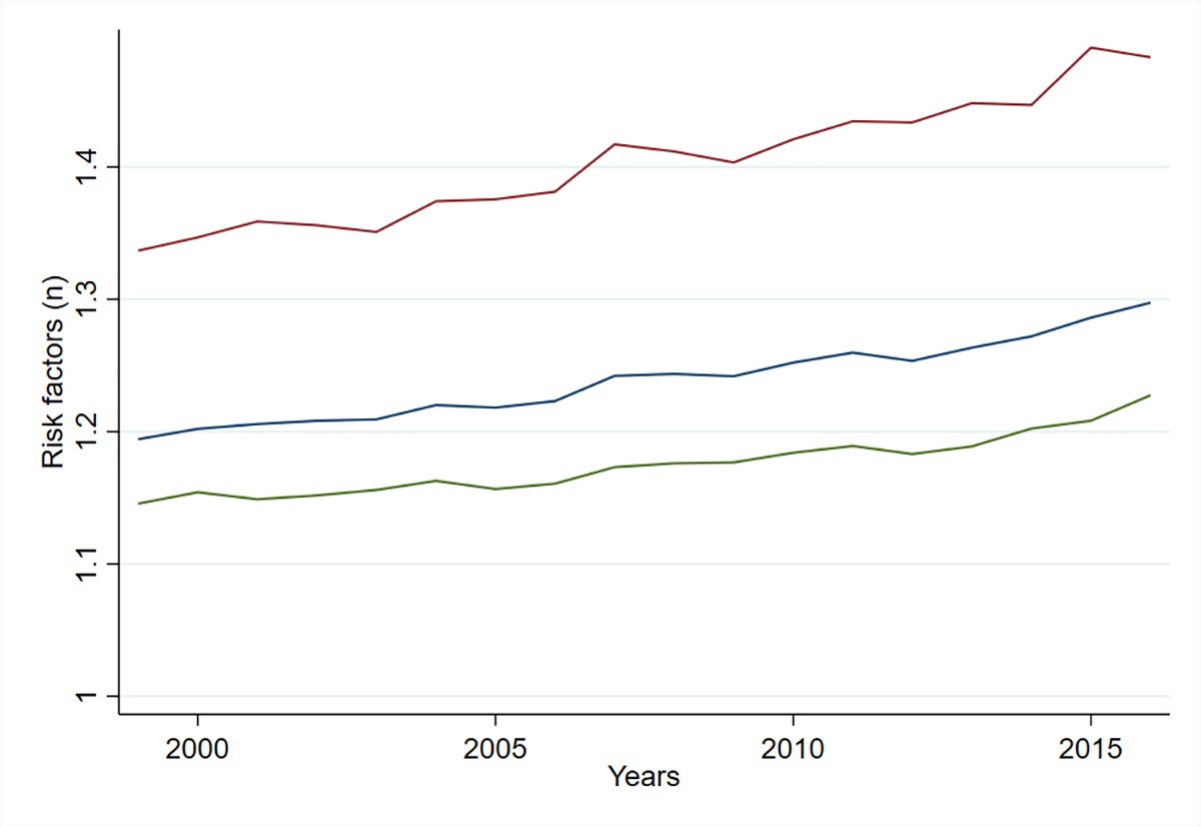
Mean number of selected risk factors per birth by year. All births (blue). Vaginal births (green). CS births (red). CS, cesarean section.

When assessed as single risk factors, and as single risk factors in combination with any other risk factor, the proportion of births with the risk factors nulliparous ≥35 years, multiparous ≥35 years, gestational diabetes, previous CS, and ART increased during the study period. The proportion of births with the risk factors hypertensive disorders and multiple births decreased, while the proportion with pregestational diabetes remained stable (Fig 3). The mean number of selected risk factors per birth increased over time for all births, vaginal births, and CS births by 9.2%, 7.0%, and 10.5%, respectively (Fig 4).

When we compared 1999 and 2016, we found that the proportion of births with 0 risk factors decreased from 74.3% to 64.9%, but the corresponding proportion of CS births in this group increased from 8.6% to 10.0% (+16.3%) (Table 2). The proportion of births with 1 risk factor increased from 21.3% in 1999 to 26.3% in 2016 (+23.5%), while the proportions of CS births in this group increased from 21.9% to 22.4% (+2.3%). Finally, the proportion of births with >1 risk factor increased from 4.5% to 8.8% (+95.6%) from 1999 to 2016, and the proportion of CS births in this group remained at 42.5%, with a peak of 47.1% in 2007.

**Table 2:** Observed number of total births, vaginal births, and CS births in 1999 and 2016.

	Year 1999 n (%)		Year 2016 n (%)	
**Unstratified**				
**Total births**	58,650		59,130	
	CS	Vaginal	CS	Vaginal
	7,571 (*12*.*9*)	51,079	9,521 (*16*.*1*)	49,609
**Stratified**				
**Births with 0 selected risk factors**	43,563 (*74*.*3*)		38,391 (*64*.*9*)	
	CS	Vaginal	CS	Vaginal
	3,729 (*8*.*6*)	39,834	3,842 (*10*.*0*)	34,549
**Births with 1 selected risk factor**	12,477 (*21*.*3*)		15,549 (*26*.*3*)	
	CS	Vaginal	CS	Vaginal
	2,733 (*21*.*9*)	9,744	3,474 (*22*.*4*)	12,074
**Births with >1 selected risk factor**	2,610 (*4*.*5*)		5,190 (*8*.*8*)	
	CS	Vaginal	CS	Vaginal
	1,109 (*42*.*5*)	1,501	2,204 (*42*.*5*)	2,986

CS, cesarean section.

**Fig 5:**
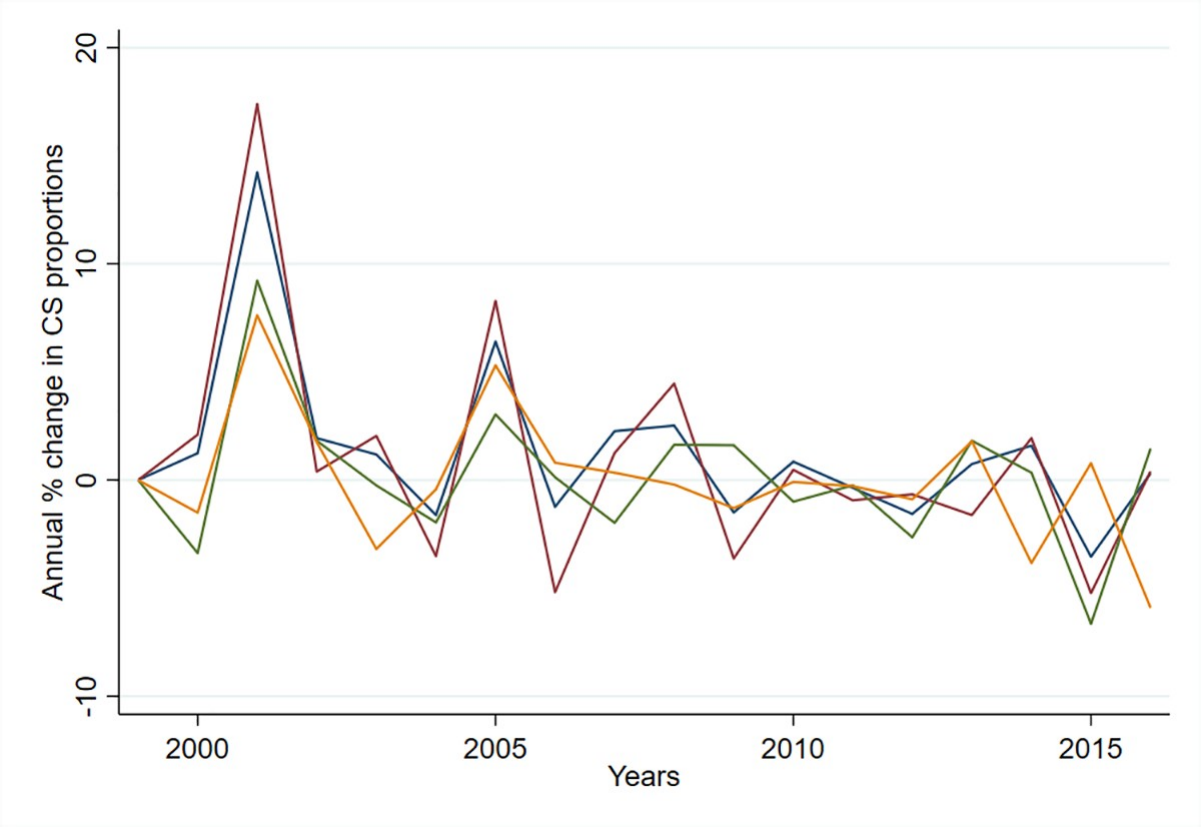
Year-to-year percent change in proportions of observed CS births overall and in stratified groups. All CS births (blue). CS births with none of the selected risk factors (red). CS births with 1 risk factor (green). CS births with >1 risk factor (yellow). CS, cesarean section.

The year-to-year percent change in CS shows the largest positive percent change from 2000 to 2001 (+14.2%) and from 2004 to 2005 (+6.4%), with more restricted or negative change dominating since 2008 (Fig 5 and S1 Table).

The proportion of CS births in 1999 was 12.9%. A crude prediction of CS births for 2016, without taking changes in maternal population during the study period into account, is number of births in 2016 * 0.129 = 7,628 (Table 3). In 2016, there were 9,521 CS births; this results in an excess of 1,893 CS births. To take the selected maternal risk factors into account, we stratified the maternal population into 0, 1, and >1 risk factors. The distribution of the maternal population in 1999 was 74.3%, 21.3%, and 4.5% for 0, 1, and >1 risk factors, respectively. This distribution changed to 64.9%, 26.3%, and 8.8% for 0, 1, and >1 risk factors in 2016. The CS proportions in 1999 were 8.6%, 21.9%, and 42.5% for 0, 1, and >1 risk factors, respectively. Taking the change in maternal population into account, one would expect an increased number of CS births for 2016, specifically 3,302, 3,405, and 2,206 CS births for 0, 1, and >1 risk factors, respectively, which sums to 8,913. By considering this change in maternal population size, the number of excess CS births is reduced to 608. The reduction in prediction error is 1,285 CS births, or 67.9% from crude to stratified prediction model. The largest increase in excess births was seen in the group of births with none of the selected risk factors (+16.4%).

**Table 3:** Difference in predicted and observed values of CS births in 2016 based on proportions in 1999.

	Predicted number of CS in 2016 based on 1999 CS proportions	Observed number of CS in 2016	Excess CS births* N (% change)
**Unstratified**			
**Total births**	7,628	9,521	1,893 (*24*.*8*)
**Stratified**			
**Births with 0 selected risk factors**	3,302	3,842	540 (*16*.*4*)
**Births with 1 selected risk factor**	3,405	3,475	70 (*2*.*1*)
**Births with >1 selected risk factor**	2,206	2,204	−2 (*−0*.*1*)
**Total number**	8,913	9,521	608 (*8*.*0*)

CS, cesarean section.

*Excess CS births = observed CS − predicted CS.

There was no uniform trend of CS among the different risk factors, either when assessed as single risk factors or when assessed in combination with any other selected risk factor (Fig 3).

**Fig 6:**
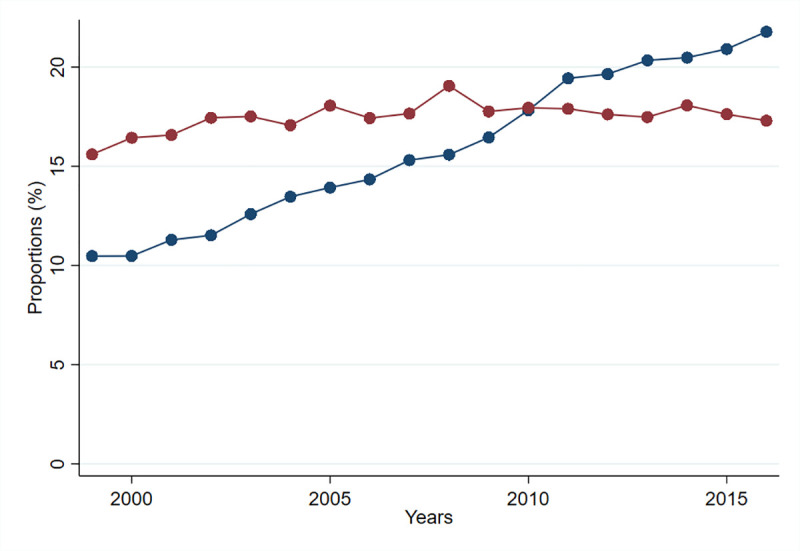
Proportion of induced labor and CS births among induced labors by year, 1999–2016. The blue line shows the proportion of births that were induced; the red line shows the proportion of CS in births that were induced. CS, cesarean section.

The proportion of induced labors doubled from 1999 to 2016, with a gradual increase every year, from 10.5% to 21.8%. The proportion of CS births among induced labors increased from 15.6% to 17.3% (+10.9%), with a peak of 19.1% in 2008 (Fig 6 and S2 Table).

## Discussion

### Main findings

The proportion of CS births in Norway increased from 12.9% in 1999 to 16.1% in 2016, an increase of 24.8%. From 2005 till study end, the proportion of CS births remained stable, while the proportion of births with selected risk factors continued to increase. Two-thirds of the excess CS births observed in 2016 compared to 1999 were associated with increases in the proportion of the population with the selected risk factors. Stratifying births depending on number of risk factors showed that the proportion of births with one of the risk factors increased by 23.5%, and the proportion of births with >1 risk factor increased by 95.6%. The largest increase in excess CS births in 2016 was observed among women with none of the selected risk factors.

Our study is one of few to assess the impact of a combination of maternal risk factors for CS over time. What our study adds to existing research is to show that Norway as a country is experiencing the international trend of an increasing population with risk factors, but this has not translated into a corresponding rise in proportions of CS at national level. On the contrary, we observed that proportions of CS births were stable from 2005 and onward. The sharp increase in proportions of CS from 2000 to 2001 coincided with the publication of the Term Breech Trial [25], which concluded that elective CS is more favorable to vaginal birth for term fetus in breech presentation. Norway is one of few countries in the Western world to practice planned vaginal delivery for selected women with fetus in breech presentation. It has been estimated that about one-third of the increase in CS proportions observed in this period is due to the influence of the Term Breech Trial, while the remaining increase could be due to a general lower threshold for performing CS [26]. Despite the steady increase in the mean number of risk factors for both vaginal and CS births over time, there was little increase in the proportion of CS in births with the selected risk factors. Instead, the moderate rise in proportions of CS at the national level may indicate that the Norwegian maternal health system, for several reasons, has not been influenced by increasing CS rates seen elsewhere in the world. In accordance with obstetrical guidelines, Norwegian clinicians seem to have practiced a conservative CS policy throughout the study period for women with known risk factors.

Obstetric care in Norway has responded to the increasing proportion of births among women with risk factors for CS by increasing the number of induced labors. The proportion of induced labors doubled over the study period, while the proportion of CS in induced labors increased by just 10.9%. This may indicate that careful selection of whom to induce at what time does not necessarily lead to an increase in the proportion of CS births, although international debate continues on whether induced labor increases the likelihood of CS [27–29]. It is important to closely monitor increases in induced labor in Norway and in many high-income countries, since it is an intervention that can lead to several maternal and newborn complications [30]. The proportion of women with induced labor was made a national quality indicator in 2016 [31], but no maximum rate was put forward and no policy has been implemented in trying to stall the increase. In addition, Norway has maintained the use of OVD, increasing from 7.6% to 10.4% over the study period, mainly in the form of vacuum extraction, as a possible alternative to CS. Several low- and middle-income countries have seen a decline in rates of OVD in periods where CS rates rose sharply [32]. Although there has been no change in protocol for the use of OVD in Norway in the study period, the observed increase could be associated with the increase in maternal age and use of epidural, both associated with an increased likelihood of OVD [33].

The 7-fold increase in gestational diabetes we observed in our study can be explained by an actual increase due to immigration from high-endemic countries, increased maternal age, and changes in lifestyle [12], but also to increased awareness of the diagnosis and screening practices [34], although national screening criteria did not change [35–37]. The observed decrease in hypertensive disorders is in accordance with observations from other high-income countries [38]. The same decreasing trend was seen in multiple births, where the reduction may be associated with protocols for ART, in which the insertion of two embryos was replaced by one in 2004/2005 [39].

Although the proportion of CS births increased during the study period, Norway has one of the lowest CS rates among high-income countries, together with the other Nordic countries (except Denmark) and the Netherlands, at 16.1% to 18.2% [40]. Our finding that two-thirds of the excess number of CS births observed at study end was associated with an increase in the size of the population with maternal risk factors does not correspond with other studies assessing the impact of maternal factors on CS. Studies from Canada, Australia, and the United States found that changes in maternal risk profiles did not account for the observed changes in CS rates [41–43]. This discrepancy is not surprising since CS rates in these countries have increased to a much larger extent than in Norway and indicates that something other than maternal risk factors is driving the increase in CS births in these countries. The results of this study may therefore only be generalizable to countries with a public health system and with general low interventions rates, but the results should also be of interest to countries who are intent on investigating their CS rates. It is interesting that the highest percentage of excess CS births in 2016 were in births without the selected risk factors. This is not a homogenous group but consists of women <35 years with no risk factors or fetal, pregnancy-related, and/or maternal factors not included in the study. Yet, the group only constitute 540 excess CS births in 2016 compared to 1999.

When considering why the overall proportion of CS births has remained low in Norway, and why the proportion of CS in births with the selected risk factors has remained stable, the organization of the country’s maternal healthcare system should be considered. First, while obstetricians have the overall medical responsibility for women with risk factors, midwives are the ones who accompany women during labor. Norwegian midwives work with a high grade of autonomy and in close collaboration with obstetricians, and the division of work is well accepted by both parties [44]. Existing research supports the idea that the care and involvement of midwives lead to fewer interventions and a higher rate of spontaneous vaginal birth compared to women cared for by doctors [45,46]. Second, Norway introduced national guidelines for obstetric care as early as 1995. These guidelines were then further elaborated into institutional guidelines. The World Health Organization strongly recommends the use of guidelines to reduce unnecessary CS [47], although studies have found that, as a stand-alone measure, guidelines are not effective in reducing CS rates [48]. General efforts to reduce the likelihood of CS among all women are included in Norwegian national guidelines. The requirement that all women should have one-to-one care by an appointed midwife during active labor [13] has been found to improve maternal and newborn outcomes; more specifically, it has reduced the likelihood of CS [49]. Third, the Norwegian maternal healthcare system invests in measures to reduce repeat CS. Finland and Norway have the highest proportions of vaginal birth after CS internationally, at 55% and 45% [40], respectively, in contrast to the US and Australia, at 12.4% [50] and 14% [51], respectively. Although additional resources are needed to offer vaginal birth after CS, large differences between countries with similar healthcare expenditures indicate that obstetric culture plays a role [52]. Women with a previous traumatic birth experience are routinely offered debriefing postpartum and counseling during subsequent pregnancy [20], and they are more frequently offered induced labor at term. Fourth, the Norwegian system provides no individual economic benefit for doctors to perform CS, which is in line with The International Federation of Gynecology and Obstetrics recommendation on how to reduce unnecessary CS [53].

### Strengths and limitations

The NMBR is a well-established registry that has been collecting information on women and newborns in Norway for more than 50 years. The database is comprehensive, and the total proportion of missing observations in our dataset was very low. Several validation studies have concluded that NMBR data are of high quality [54–57], apart from underreporting of severe maternal complications in one study [58]. A weakness of the study is that we included maternal age as a binary variable with a cutoff at ≥35 years, while the likelihood of emergency CS has been shown to display a linear association from an early age [59]. With an increase in the age groups ≥30 years during the study period, this cut-off can lead to an underestimation of the relationship between maternal risk factors and increases in CS rates. Another weakness is that we were not able to include obesity, previous traumatic vaginal delivery, mental disorders, and birth anxiety, known maternal risk factors for CS. We also did not take into consideration the increase in births to immigrant mothers, a group found to have higher likelihood of emergency CS compared to ethnic Norwegian mothers [60,61]. These limitations may have led to an underestimation of the association between the selected risk factors and the increase in CS births from 1999 to 2016. Moreover, comparing only the first and the last year of the study period removes nuances in year-to-year changes. We did not explore which of the selected risk factors had the greatest influence on the change in CS proportions, which could have provided additional information of relevance for clinicians.

With a steady increase in the mean number of maternal risk factors for CS per birth for both vaginal and CS births, and with an increase in the proportion of women with these risk factors, the maternal healthcare system must adapt to accommodate women with an increased need of follow-up and possible interventions during pregnancy and labor. The system of selecting women to the appropriate level of care and continuity of care are strategies that could improve outcomes for women with risk factors for CS and avoid unnecessary interventions. Further exploration of what combination of risk factors contribute the most to proportions of CS would be of clinical interest. So would a study that identified the main risk factors in the group with none of the selected risk factors in this study.

In conclusion, from 1999 to 2016, the proportion of CS in Norway increased from 12.9% to 16.1%, with minor changes from 2005. Throughout the study period, 5 out of 8 selected risk factors increased, while the proportions of CS births among women with these risk factors remained stable. We observed a possible association between population increase in the proportion of births with the selected risk factors and the excess CS births observed in 2016 compared to 1999. The stable CS rate from 2005 and the increasing size of risk population may indicate that Norwegian maternal health practitioners have managed to balance the care of an increasingly morbid population without following the international increase in CS rates.

## Supporting information

S1 STROBE Checklist STROBE, Strengthening the Reporting of Observational Studies in Epidemiology. (DOCX)

S1 Personal Communication Permission to use information on the handling of deviant information in the Norwegian birth registry. (PDF)

S1 Table Annual change in CS births. Year-by-year percent change in proportions of observed CS births overall and in stratified groups, 1999–2016. CS, cesarean section.
(DOCX)

S2 Table Induction of labor and CS births. Proportion of induction of labor and CS births among induced births by year, 1999–2016. CS, cesarean section.
(DOCX)

## Abbreviations

ART: assisted reproductive technology
CS: cesarean section
HELLP: syndrome, hemolysis, elevated liver enzymes, low platelet count
NMBR: Norwegian Medical Birth Registry
OVD: operative vaginal deliveries
