# Supplementary material for: Changes in maternal risk factors and their association with changes in cesarean sections in Norway between 1999 and 2016: A descriptive population-based registry study
Source: PLoS Med. 2021 Sep 3;18(9):e1003764. doi: 10.1371/journal.pmed.1003764 (PMC8452082; doi:10.1371/journal.pmed.1003764)
Supplement: S1 Personal Communication — (PDF) [file pmed.1003764.s002.pdf]

Bergen 02.06.2021

To the editor of PLOS Medicine

I hereby give my permission to reference the Personal Communication between the first author and myself on 1. November 2019 in the manuscript entitled "Changes in maternal risk factors and their association with observed changes in the proportion of caesarean section: A descriptive population-based registry study from Norway 1999-2016 (PMEDICINE-D-21-00861R1).

I approve to use of the following line in the manuscript: "Predetermined violations of biological plausibility in the online form are handled by the operational staff at the NMBR".

Yours sincerely,

Henriette Hagen Hansen

Henriette Hagen Hansen

Advisor,

The Norwegian Medical Birth Registry

Avdeling for helsedatamottak, Medisinsk fødselsregister

Folkehelseinstituttet

Postadresse: Postboks 973 Sentrum; 5808 Bergen

Besøksadresse: Zander Kaaes gate 7

Mob: 00 47 945 27 643

www.fhi.no

henriette.hagenhansen@fhi.no
