# Supplementary material for: Changes in maternal risk factors and their association with changes in cesarean sections in Norway between 1999 and 2016: A descriptive population-based registry study
Source: PLoS Med. 2021 Sep 3;18(9):e1003764. doi: 10.1371/journal.pmed.1003764 (PMC8452082; doi:10.1371/journal.pmed.1003764)
Supplement: S1 Table — Year-by-year percent change in proportions of observed CS births overall and in stratified groups, 1999–2016. CS, cesarean section. (DOCX) [file pmed.1003764.s003.docx]

**S1 Table.** **Year-to year percent change in proportions of observed caesarean section births overall and in stratified groups.**

|  | **1999** | **2000** | **2001** | **2002** | **2003** | **2004** | **2005** | **2006** | **2007** | **2008** | **2009** | **2010** | **2011** | **2012** | **2013** | **2014** | **2015** | **2016** |
| --- | --- | --- | --- | --- | --- | --- | --- | --- | --- | --- | --- | --- | --- | --- | --- | --- | --- | --- |
| **Unstratified** |  |  |  |  |  |  |  |  |  |  |  |  |  |  |  |  |  |  |
| **Total CS** | Ref. | 1.24 | 14.23 | 1.94 | 1.18 | -1.62 | 6.40 | -1.24 | 2.26 | 2.52 | -1.50 | 0.85 | -0.36 | -1.57 | 0.74 | 1.59 | -3.55 | 0.31 |
| **Stratified** |  |  |  |  |  |  |  |  |  |  |  |  |  |  |  |  |  |  |
| **Births with 0 selected risk factors** | Ref. | 2.10 | 17.39 | 0.39 | 2.04 | -3.52 | 8.28 | -5.19 | 1.25 | 4.46 | -3.63 | 0.47 | -0.94 | -0.66 | -1.62 | 1.94 | -5.23 | 0.40 |
| **Births with 1 selected risk factor** | Ref. | -3.38 | 9.22 | 1.82 | -0.25 | -1.96 | 3.04 | 0.13 | -1.98 | 1.63 | 1.61 | -1.0 | -0.25 | -2.65 | 1.82 | 0.34 | -6.65 | 1.45 |
| **Births with >1 selected risk factor** | Ref. | -1.51 | 7.62 | 1.75 | -3.19 | -0.43 | 5.30 | 0.80 | 0.34 | -0.21 | -1.30 | -0.09 | -0.28 | -0.89 | 1.79 | -3.84 | 0.78 | -5.91 |

CS; caesarean section
