# Supplementary material for: Changes in maternal risk factors and their association with changes in cesarean sections in Norway between 1999 and 2016: A descriptive population-based registry study
Source: PLoS Med. 2021 Sep 3;18(9):e1003764. doi: 10.1371/journal.pmed.1003764 (PMC8452082; doi:10.1371/journal.pmed.1003764)
Supplement: S2 Table — Proportion of induction of labor and CS births among induced births by year, 1999–2016. CS, cesarean section. (DOCX) [file pmed.1003764.s004.docx]

**S2 Table. Proportion of induced labour and CS births among induced labours by year, 1999-2016.**

| **Years** | **Total *(row total %)*** | **CS *(%)*** |
| --- | --- | --- |
| 1999 | 6 142 *(10.5)* | 958 *(15.6)* |
| 2000 | 6 139 *(10.5)* | 1 009 *(16.4)* |
| 2001 | 6 341 *(11.3)* | 1 051 *(16.6)* |
| 2002 | 6 352 *(11.5)* | 1 108 *(17.4)* |
| 2003 | 7 086 *(12.6)* | 1 241 *(17.5)* |
| 2004 | 7 635 *(13.5)* | 1 303 *(17.1)* |
| 2005 | 7 858 *(13.9)* | 1 419 *(18.1)* |
| 2006 | 8 348 *(14.3)* | 1 455 *(17.4)* |
| 2007 | 8 903 (*15.3)* | 1 572 *(17.7)* |
| 2008 | 9 409 *(15.6)* | 1 793 *(19.1)* |
| 2009 | 10 184 *(16.5)* | 1 809 *(17.8)* |
| 2010 | 10 970 (17.8) | 1 969 *(18.0)* |
| 2011 | 11 723 *(19.4)* | 2 098 *(17.9)* |
| 2012 | 11 855 *(19.7)* | 2 088 *(17.6)* |
| 2013 | 11 999 *(20.3)* | 2 097 *(17.5)* |
| 2014 | 12 098 *(20.5)* | 2 186 *(18.1)* |
| 2015 | 12 326 *(20.9)* | 2 172 *(17.6)* |
| 2016 | 12 879 *(21.8)* | 2 228 *(17.3)* |
| **Total** | 168 247 *(16.0)* | 29 556 *(17.6)* |

CS; caesarean section
